# Synaptopathy in Guinea Pigs Induced by Noise Mimicking Human Experience and Associated Changes in Auditory Signal Processing

**DOI:** 10.3389/fnins.2022.935371

**Published:** 2022-07-06

**Authors:** Li Xia, Sara Ripley, Zhenhua Jiang, Xue Yin, Zhiping Yu, Steve J. Aiken, Jian Wang

**Affiliations:** ^1^Department of Otolaryngology-Head and Neck Surgery, Mianyang Central Hospital, School of Medicine, University of Electronic Science and Technology of China, Mianyang, China; ^2^School of Communication Sciences and Disorders, Dalhousie University, Halifax, NS, Canada

**Keywords:** temporal processing, coding-in-noise deficit, cochlear efferent, fluctuation profile, Guinea pigs, noise-induced synaptopathy

## Abstract

Noise induced synaptopathy (NIS) has been researched extensively since a large amount of synaptic loss without permanent threshold shift (PTS) was found in CBA mice after a brief noise exposure. However, efforts to translate these results to humans have met with little success—and might not be possible since noise exposure used in laboratory animals is generally different from what is experienced by human subjects in real life. An additional problem is a lack of morphological data and reliable functional methods to quantify loss of afferent synapses in humans. Based on evidence for disproportionate synaptic loss for auditory nerve fibers (ANFs) with low spontaneous rates (LSR), coding-in-noise deficits (CIND) have been speculated to be the major difficulty associated with NIS without PTS. However, no robust evidence for this is available in humans or animals. This has led to a re-examination of the role of LSR ANFs in signal coding in high-level noise. The fluctuation profile model has been proposed to support a role for high-SR ANFs in the coding of high-level noise in combination with efferent control of cochlear gain. This study aimed to induce NIS by a low-level, intermittent noise exposure mimicking what is experienced in human life and examined the impact of the NIS on temporal processing under masking. It also evaluated the role of temporal fluctuation in evoking efferent feedback and the effects of NIS on this feedback.

## Introduction

The concept of noise induced hearing loss (NIHL) has been greatly enriched by the discovery of massive synaptic loss in cochleae without permanent threshold shifts (PTS) in animal studies (Kujawa and Liberman, 2009; Moser et al., 2013; Starr and Rance, 2015; Moser and Starr, 2016; Song et al., 2016; Kaur et al., 2019; Kim et al., 2019; Liu et al., 2019). Noise-induced synaptopathy (NIS) without PTS and noise-induced hidden hearing loss have become hot topics in hearing research since then. Due to the difficulty obtaining morphological data for cochlear synaptic loss cause by noise in humans, animal data has been used to interpret or predict NIS in human subjects. However, this translation has not been validated since the noise exposures used in the animal studies are mostly brief (e.g., 2 h) exposures at the maximum level that does not cause PTS (100–106 dB SPL). Such noise is not likely to be experienced by human subjects, for which traffic noise (Munzel and Sorensen, 2017; Nieuwenhuijsen et al., 2017; Zare Sakhvidi et al., 2018; Munzel et al., 2020), recreational noise (Ivory et al., 2014; Fulbright et al., 2017), noise in industrial settings (Stucken and Hong, 2014; Lie et al., 2016) and in military activity (Pfannenstiel, 2014; Nakashima and Farinaccio, 2015) are the major concerns. Except for military noise, these other common noise types do not have ongoing levels over 100 dB SPL. In industrial settings, which used to be major sources of NIHL, noise levels received by human ears rarely exceed 90 dB SPL under current safety regulations. The noise from traffic and recreational events may frequently peak at very high levels, but only lasts for very short periods of time (Jagniatinskisa et al., 2017; Oiamo et al., 2017). On the other hand, the noise in all of the above situations is amplitude modulated (Barlow and Castilla-Sanchez, 2012; Masullo et al., 2016), not stationary like the noise used in the laboratory studies. Moreover, noise-induced damage of human hearing accumulates over many years in which noise exposure is intermittent. Therefore, synaptic damage by the real-life noise is likely different from the damage created by the noise used in laboratory studies.

Functionally, NIS without PTS is associated with the concept of noise induced hidden hearing loss (NIHHL). Based on a selective loss of afferent synapses innervating auditory nerve fibers (ANFs) with low spontaneous rates (LSR) in two animal studies (Furman et al., 2013; Song et al., 2016) and the theory that LSR ANFs are necessary for signal coding in high level background noise, coding-in-noise deficits (CIND) or deficits of hearing in noise (DHIN) have been considered to be the major hearing problem associated with NIHHL (Plack et al., 2014; Le Prell and Clavier, 2017; Liberman, 2017; Liberman and Kujawa, 2017; Chen et al., 2019a; Huet et al., 2019; Kohrman et al., 2020). However, there is no reliable evidence supporting the existence of CIND in either animals or human subjects. The equivocal results have challenged the proposed unique role of LSR ANFs in coding high-level sounds and led to a reconsideration of high SR (HSR) ANFs in high-level signal coding. For example, the recently proposed fluctuation profile model suggests that high-level sounds are mainly coded by HSR ANFs (Carney, 2018). Interestingly, this model posits that efferent control of cochlear gain is part of mechanism and is sensitive to temporal fluctuation of auditory input although no evidence for this is reported.

In the present study, we aimed to (1) examine NIS without PTS by using a temporarily modulated noise with a long-term equivalent (Leq) sound level of 90 dB SPL, presented intermittently over a month to mimic noise exposures in human subjects, (2) to determine whether the resulting NIS impacts the ability of subjects to use temporal cues for coding masked signals, and (3) to evaluate the role of temporal fluctuations in contralateral suppression of the compound action potential (CAP) and determine whether this is affected by NIS.

## Materials and Methods

### Outline of Subjects and Main Procedures

A total of 20 adult albino guinea pigs (Hartley) were obtained from Charles River, Canada for this study; 10 in the control and the noise groups, respectively. After the animals were recruited (at the age of 1.5–2.5 months), their external ears were checked for abnormalities. The animals were then tested with frequency-specific auditory brainstem responses (ABR) to ensure normal hearing sensitivity. In this baseline test, the envelope following response (EFR) was also measured in the far-field. Following the baseline test, the animals in the noise group were subjected to a noise exposure over a one-month period. One-month after the noise exposure or two months after the baseline test, ABR and EFR were repeated on the animals in each group, followed by a set of near-field recordings (from the round window), including the transient CAP and in response to amplitude modulation (AM, or near-field EFR—nfEFR). Following the terminal evaluation, the animals were sacrificed, and their cochleae were harvested for a morphological evaluation of ribbon synapse count. All of the procedures were approved by the University Committee of Laboratory Animals (protocol# 20-024).

### Noise Exposure

Multi-talker babble was modified to be more suitable for Guinea pig hearing by shifting it to 2-16 kHz using a noise vocoder approach implemented in Matlab (Dorman et al., 1997) (see detail in Supplementary Material). The frequency-shifted multi-talker noise was presented in a sound booth *via* a four-speaker array (Pyramid TW-67 Super Tweeters; Brooklyn, NY, United States), which was suspended 40 cm above the sound booth floor. Throughout the noise exposure, the animals were awake and unrestrained in a metal wire cage inside the sound booth with free access to water and food (Chen et al., 2019b; Fan et al., 2020; Zhang et al., 2020). The animals were exposed to the noise presented an Leq of 90 dB SPL for 8–12 h per day. This was done on every other day to allow for a day of rest following each episode of noise exposure. The total duration of the noise exposure was 122 h, making the total energy of the noise roughly equal to the 2-h exposure at 106 dB SPL that has been used in previous studies in Guinea pigs (Chen et al., 2019b; Fan et al., 2020; Zhang et al., 2020).

### Auditory Brainstem Response and Envelop Following Responses

All electrophysiological evaluations were performed in an electromagnetically shielded sound booth. Guinea pigs were anesthetized with a mixture of ketamine and xylazine by intraperitoneal injection for the ABR and EFR baseline tests. The initial dose was 40 and 10 mg/kg for ketamine and xylazine, respectively, and 1/3 of the initial dose was added as needed to maintain the anesthesia as needed (judged by the toe-pinching reflex) when the test was exceptionally longer than 1 h. Throughout the experiment, the body temperature of the animal was kept at 38°C with a thermostatic heating pad. In the terminal evaluation, all of the tests were completed with the animals under urethane (i.p., 1.5 g/kg).

An auditory signal processing station (RZ6) from Tucker-Davis Technologies (TDT System III; Alachua, FL, United States) was used to generate the signals for auditory stimulation and to record the biological responses. The acoustic signals for all the auditory responses were delivered in open field *via* a broadband speaker (FT28D, Fostex). Maskers for EFR recording were also delivered in open field *via* an additional FT28D speaker.

Both ABR and EFR were recorded with three subdermal electrodes, with the recording electrode inserted at the vertex and the reference and grounding electrodes positioned posterior to the external auditory canals. The biological signals picked up by the electrodes were sent to an RA16PA preamplifier, which amplified the signal 20 times.

Auditory brainstem response (ABR) was evoked by 10-ms tone bursts (tone bursts) with a rise/fall time of 0.5 ms. The tone bursts were presented 21.1/s for the ABR and ABR thresholds were measured from 1 to 32 kHz in octave steps. For each trial, the response was averaged 1000 times; fewer averages were collected if a clear response was visible. At each frequency, tone bursts were presented in a descending sequence from 90 dB SPL toward threshold, which was defined as the lowest sound level at which a repeatable Wave-III was visible.

Envelop following responses (EFR) was evaluated in response to 16 kHz AM tones that were presented at a moderately high level of 75 dB SPL. The AM tones were presented in a sweeping pattern, and they had a duration of 500 ms and a rise/fall time of 5 ms. The modulation frequency (MF) was initially set from 113 to 1513 Hz in 100 Hz steps to get a TMTF, which was evaluated at two modulation depths (MD): 30 and 60%. The EFR was sampled at 24.414 kHz over a 500-ms time window to cover the length of the stimuli. The response of the first 50 ms was set to zero to avoid the impact of the onset response. In each trial, EFR was averaged 50 times before it was converted into the frequency domain by a Fast Fourier Transformation. The spectral peak at each MF was measured in dB as the phase-locked response to the MFs.

Following the testing in quiet, the effect of the masker on EFR was evaluated at the best MF (best modulation frequency), i.e., the MF at which the greatest response occurred by each of the two maskers: one was a high pass filtered white noise with a cutoff at 4 kHz (the stationary masker) and the other was the multi-talker noise used for the noise exposure (the masker with fluctuation). Each masker was played at 75 dB SPL [yielding a 0 dB signal-to-noise ratio (signal-to-noise ratio)].

To mitigate the impact of random changes in EFR with time, each masked EFR was sandwiched by two control recordings (without masking). This strategy was also used for the recording of the nfEFR. The two EFRs without masking were averaged and the effect of masking was calculated as the difference of magnitude in dB between the EFRs with and without masking.

### Compound Action Potential and Near-Field Envelop Following Responses Recording From Round Window

Under anesthesia *via* urethane (i.p., 1.5 g/kg), a silver ball electrode was placed on the round window membrane after the mastoid was surgically opened. To secure the electrode in place, the silver wire was fixed to the mastoid with dental cement. The other end of the silver wire as well as the reference and grounding electrodes were connected to the preamplifier and then to the TDT system, exactly the same way as for the ABR and EFR recordings. A plastic tube was embedded in the dental cement to provide ventilation of the middle ear, preventing the buildup of negative pressure. During the surgery and recording, the animal was placed on a thermostatic heating pad to maintain a body temperature of 38°C. The nfEFR was measured and analyzed the same way as the scalp EFR, except that the number of averages in each trial was 25 instead of 50.

The transient CAP was evoked by a 16 kHz tone burst with 2-ms duration (0.5 ms rise/fall) from 90–10 dB SPL to obtain I/O functions. The stimuli were delivered in open field *via* a FT28D speaker. The effect of contralateral suppression (CS) was observed in CAP evoked by 16 kHz tone bursts. The CS signal was delivered in closed field *via* a MF-1 speaker with tubing. Three types of signals were used as CS stimuli: (1) 16 kHz tone without modulation, (2) 16 kHz tone sinusoidally modulated by 93 Hz at 30% MD, and (3) at 60% MD. With each type of CS signal, the CS effect was observed at three CS levels: 75-, 63-, and 51-dB SPL. Therefore, the CS effect was observed under 9 conditions (3 types at 3 levels).

Similar to the masking effect test, each CAP with CS was sandwiched by two records without CS to mitigate the impact of random variation of the CAP over time. The two controls were averaged for the calculation of the CS effect, which was the difference in the CAP with and without CS.

### Synapse Count Observation

The morphological evaluation was carried out in accordance with previously published procedures (Liu et al., 2012; Shi et al., 2013; Song et al., 2016; Chen et al., 2019b; Fan et al., 2020; Zhang et al., 2020). To begin, the cochlear tissues were dissected after being fixed with 4% paraformaldehyde in phosphate-buffered saline (PBS). They were then permeabilized with 1% Triton X-100 in PBS for 1 h, incubated in 5% goat serum in PBS for an additional 1 h, and then incubated overnight at 4°C with primary antibodies against both C-terminal binding protein 2 (CtBP2) and post-synaptic density-95 (PSD95) (mouse IgG1 to CtBP2; BD Biosciences, Franklin Lakes, NJ, United States: cat. # 612044, 1:200; mouse IgG2a to PSD95; Millipore, Billerica, MA, USE: cat. # MAB1596, 1:600). After the reaction, the tissues were washed and treated with the corresponding secondary antibodies (A21124 and A21131, respectively; Invitrogen, Carlsbad, CA, United States) at room temperature for 2 h, and then mounted on microscope slides.

A confocal laser-scanning microscope (LSM 710 META; Zeiss, Shanghai, China) with a 63 × water-immersion objective was used to obtain confocal images at specified frequency positions based on frequency-distance mapping (Viberg and Canlon, 2004). Next, image stacks were exported to ImageJ image-processing software (National Institutes of Health, Bethesda, MD, United States). In order to obtain the puncta densities, over 10 successive inner hair cells at each frequency position of the cochlea were selected to count the puncta of CtBP2 and PSD95.

### Data Analysis

The ABR and EFR were repeated at two time points (baseline and end test) in each of the control and noise group, generating 4 data sets which were labeled as ctrl-young, ctrl-old in the control group, and pre-noise and post-noise in the noise group. Useful data was not obtained from every subject due to unexpected recording problems. The exact sample size was specified for each test result, either by the number in the brackets in the figure legends or as stated in the figure legend.

All data in this report are presented as means ± standard error of mean (SEM). To analyze the data, the data were first evaluated for normality and equal variances. Parametric tests were performed for data passing the normality and equal variance tests, otherwise, non-parametric tests were applied. All statistics were done using SigmaPlot 14. For data with multiple factors, ANOVAs were followed by *post hoc* pairwise evaluations. P < 0.05 was used as the criterion for significance.

## Results

### Auditory Brainstem Response

**FIGURE 1:**
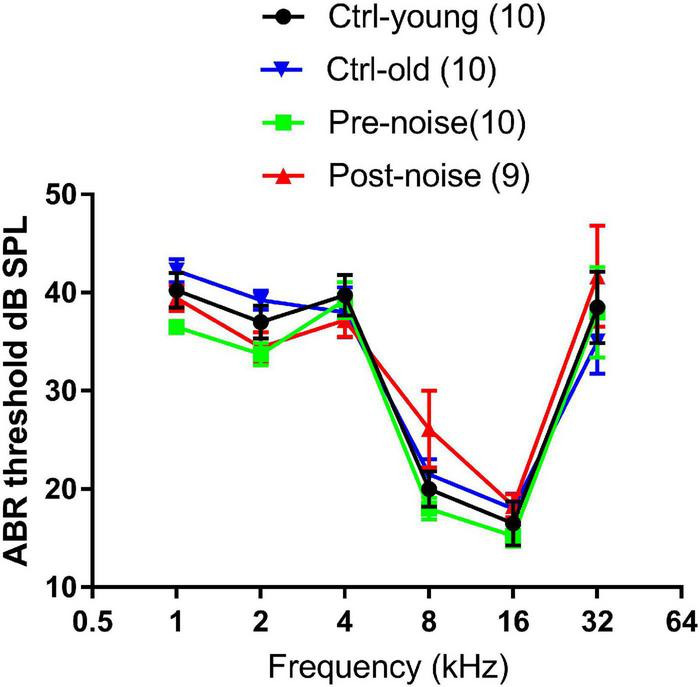
The effect of age and noise on ABR thresholds. Ctrl-young and Pre-noise were the baseline thresholds taken at 1.5–2 months of age from both the control and noise groups before the noise exposure. Ctrl-old was measured at 4–5 months of age (from the control group), which matched the age of the noise group for the ABR tested one month after the noise exposure.

The hearing status of the animals was examined with ABR in the noise group before and one month after the noise exposure to confirm that the noise exposure did not cause PTS. ABR was also tested in the control group across the times of the experiment to rule out any age-related change in auditory sensitivity. Figure 1 shows ABR thresholds tested from the two groups at two time points. The ABR-frequency curves measured at the two time points in the control group were largely overlapping, indicating that there was no age effect on the ABR threshold. This was supported by the insignificant difference (F1 = 0.712, *p* = 0.422) between the repeated tests in the two-way repeated measure (RM) ANOVA against the test and frequency. A two-way ANOVA was conducted to compare the Ctrl-old and Post-noise groups (with frequency as a co-variant) to determine whether noise exposure had any impact on thresholds. No significant effect of group was seen between the two groups (F1 = 0.156, *p* = 0.694).

### Synapse Count Observation

**FIGURE 2:**
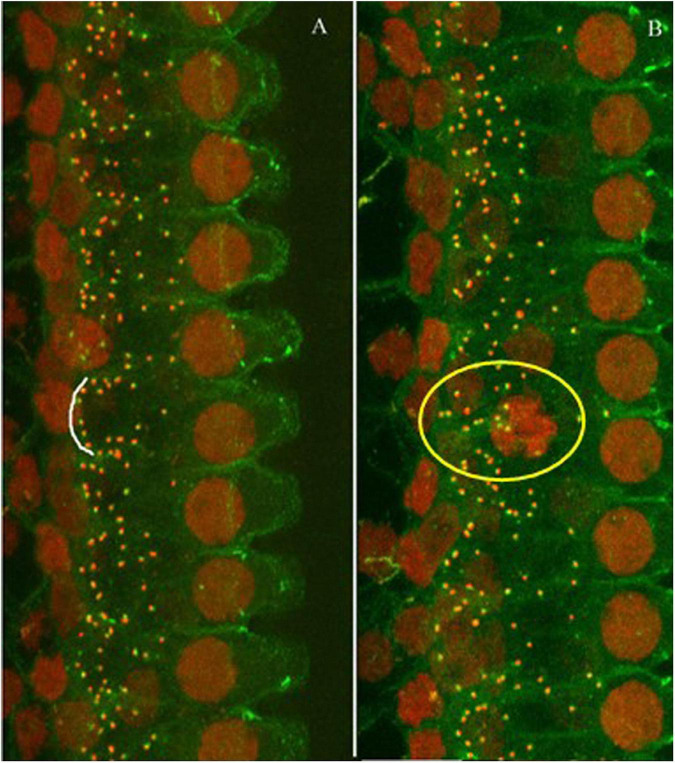
Representative images of immunostaining against CtBP2 (red dots) and PSD (green dots). **(A)** Control animal. **(B)** Noise-exposed animal (one-month post-noise exposure). The images were taken from the high frequency region of the cochlea. The distribution of the puncta was mostly alone the bottom of the IHCs as (the white arc), but less organized in the noise-exposed cochlea (see the puncta in the yellow circle).

The ribbon synapses were identified by immunohistochemistry. The densities of the synapses were compared between the groups and with the previous data to verify the amount of synaptic loss from the noise exposure used in this study. Figure 2 shows representative images of immunostaining against CtBP2 (red dots) and PSD (green dots) from both a control animal and a subject exposed to the noise (one month after). The images were taken from the high frequency region of the cochlea. The images show that the synaptic puncta are distributed mostly along the bottom of inner hair cells in the control cochlea (indicated by the curve along the bottom of an inner hair cell in Figure 2A), while the distribution is less organized, or widely distributed in the noise-exposed cochlea (as shown in the circulated area in Figure 2B).

**FIGURE 3:**
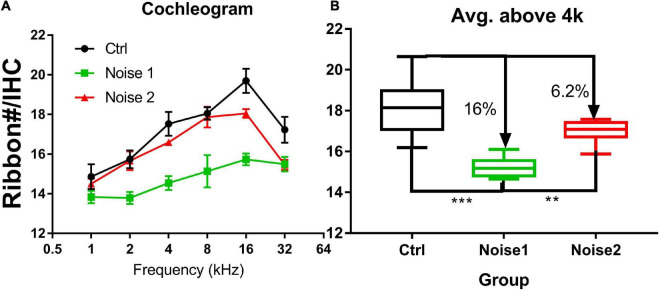
Synapse density comparison across groups (*n* = 8 in every group). **(A)** The cochleogram of synaptic density. **(B)** The averaged synaptic density in the frequency region above 4 kHz. The synapse density is calculated from ribbons (Ctbp2). Noise 1 refers to a brief noise at 106 dB SPL for 2 h [data taken from a previous study Song et al. (2016)]. Noise 2 refers to the noise exposure examined in the present study (multi-talker noise, repeated over a period of 1 month for 122 h with an Leq of roughly 90 dB SPL). The density was compared between groups. ***p < 0.001, **p < 0.01.

Figure 3 compares the ribbon densities (stained against CtBP2) across groups. The data from a previous study were taken for the synaptic counts after a brief-noise exposure at a higher level (106 dB SPL, 2 h; noise 1) (Song et al., 2016) to compare with the low-level noise (∼90 dB SPL) given periodically over one month with a roughly equal dose in the present study (122 h, noise 2). Since the ribbon puncta are mostly paired with PSDs (Figure 2), the ribbon counts were used to indicate the number of synapses. This practice is supported by previous studies, which have shown that the numbers of CtBP2 puncta and the postsynaptic puncta are similarly changed after noise damage (Maison et al., 2013; Shi et al., 2013, 2016; Wang et al., 2015). Figure 3A shows the ribbon density-frequency map (or density cochleogram). Figure 3B compares the density averaged over the frequency region above 4 kHz. This average was 18.15 ± 0.387 in the control group and 15.18 ± 0.185 in the group exposed to the brief noise (noise 1, 16% lower than control). Average density was 16.99 ± 0.12 after the long-term noise exposure (noise 2, 6.2% lower than control). A one-way ANOVA on rank (Kruskal-Wallis) showed a significant overall difference between groups (H_2_ = 19.79, *p* < 0.001). *Post hoc* pairwise tests (Dunn’s method) showed significant differences between the control and noise 1 groups (*Q* = 4.445, *p* < 0.001) and between the noise 1 and noise 2 groups (*Q* = 3.029, *p* < 0.007), but not between control and noise 2 groups (*Q* = 1.983, *p* = 0.142).

### Envelop Following Responses and Near-Field Envelop Following Responses

#### Temporal Modulation Transfer Functions

**FIGURE 4:**
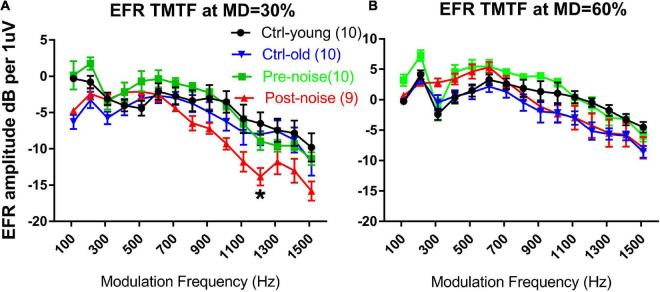
The impact of age and noise on EFR TMTFs measured in response to AM at 30% **(A)** and 60% **(B)** MD. The post-noise TMFT curve obtained with 30% MD diverged from the pre-noise and ctrl-old TMTFs. A significant difference in EFR amplitude was seen between the ctrl-old and the post-noise TMTF at 1213 Hz MF. *p < 0.05.

Both EFR and nfEFR were observed to show the impact of the noise exposure on temporal processing, and to determine whether the damage to cochlear function would be reflected in the response recorded from scalp. Figure 4 shows the impact of noise and age on the TMTF as assessed *via* EFR. TMTFs at 30% MD and 60% MD from the two groups at the two time points are given in Figures 3A,B, respectively. The TMTFs measured with 60% MD for the Ctrl-old and post-noise groups were largely overlapping at high MFs, while the TMTFs measured with 30% MD for the post-noise group diverged from the Ctrl-old TMTFs at high MFs, with the largest difference at 1213 Hz. At this MF, the difference between the groups was statistically significant (Mann-Whitney Rank Sum Test, *T* = 59, *p* = 0.013).

**FIGURE 5:**
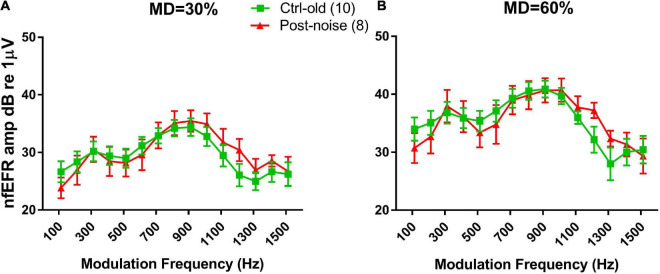
TMTFs of nfEFRs at 30% MD **(A)** and 60% MD **(B)**. The results for the two groups are largely overlapping.

However, the significant change in the EFR TMTF was not seen in the nfEFR. Figure 5 shows the nfEFR TMTFs between the groups. Since the nfEFR can only be recorded in the terminal test, they are shown only for the old age group without noise exposure (Ctrl-old) and the old age group post-noise exposure (Post-noise). Unlike the TMTFs in the far-field recording, those obtained in the near field are largely overlapping for both groups.

#### Effect of Stationary and Temporally Modulated Maskers

**FIGURE 6:**
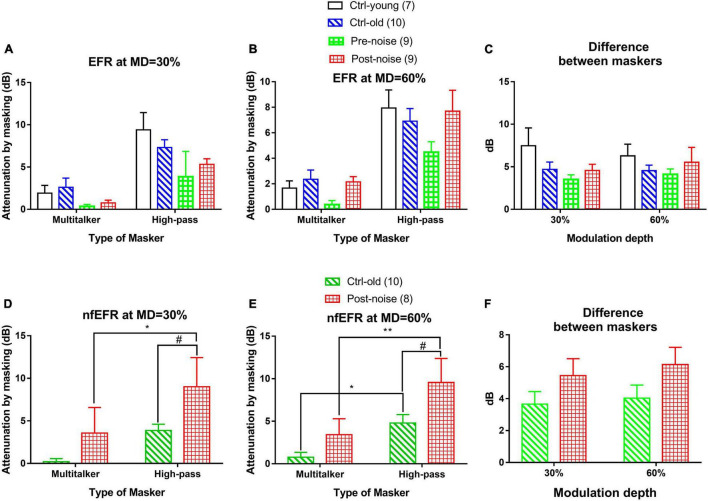
The effect of both the modulated (multi-talker) and stationary (high-pass) maskers at the two MDs [30% **(A,D)** and 60% **(B,E)**] for both the EFR **(A,B)** and nfEFR **(D,E)**, as well as the difference in the masking effect between the two maskers [**(C)** for EFR and **(F)** for nfEFR]. Overall, the high-pass noise produced more masking than the multitalker noise. No significant difference was seen between the two maskers with respect to EFR amplitude **(C)**. For the nfEFR, the HP noise resulting in a greater masking effect for the post-noise group than for the control [# in **(D,F)**]. The difference between the two maskers in nfEFR **(F)** was much larger in the noise group as seen in the two-way ANOVA. However, *post hoc* tests found no significant differences within each MD. The number of “*” symbols show the significance level of the *post hoc* comparisons within each group, while the number of “#” symbols show the significance level of the *post hoc* comparisons within each masker: one for *p* < 0.05, two for *p* < 0.01, and three for *p* < 0.001.

The impact of masker types on masking effect was observed in both EFR (Figures 6A–C) and nfEFR (Figures 6D–F) between the stationary masker and modulated multi-talker babble. The effect of each masker at the best modulation frequency of each subject was calculated as the difference of the response amplitude with and without masking, or the attenuation of the response by masking in dB. Universally, the masking effect was much larger when the stationary masker was used than when the modulated masker was used. For example, under 30% MD in the post-noise testing (Figure 6A), the effect of masking on EFR amplitude using multitalker noise was 0.753 ± 0.328 dB, while the effect of masking using the HP masker was 5.318 ± 0.66 dB. A paired *t*-test indicated that this difference was significant (*t* = 6.625, *p* < 0.001). However, the difference between the two maskers (Figure 6C) did not show much variation between the groups and between the two tests within each group. For example, the difference between the two maskers with respect to their effects on the EFR at 30% MD in the post-noise test was not significantly smaller than in the pre-noise control (3.509 ± 0.569 versus 4.564 ± 1.842, paired *t*-test: *t* = −1.185, *p* = 0.27). This negative result is inconsistent with the idea that noise-induced synaptic damage impairs signal coding in modulated maskers.

The masking to nfEFR by the two maskers were shown also at two MDs (Figures 6D,E, respectively, for 30 and 60% MD). Similar to the result in EFR, the masking effect by the high-pass noise appeared to be larger than that of multi-talker masker and the masking effect by the two maskers appeared to be larger in the Post-noise group. A two-way ANOVA was performed at each MD for the factors of group and masker type. The analysis revealed a significant effect of both masker type (F_1_ = 7.401 and *p* = 0.010 for MD = 30%, F_1_ = 15.716 and *p* < 0.001 for MD = 60%), and group (F_1_ = 6.458 and *p* = 0.016 for MD = 30%, F_1_ = 8.339 and *p* = 0.007 for MD = 60%).

*Post hoc* comparisons (Holm-Sidak method) revealed a significant effect of group within the stationary masker for both 30% MD (*t* = 2.175, *p* = 0.037) and 60% MD (*t* = 2.622, *p* = 0.013) (marked by “#” in Figures 6D,E). Further, significant effect of masker type marked by “*” was seen within the Post-noise group (*t* = 2.183, *p* = 0.036) at 30% MD, and within the Ctrl-old group (*t* = 2.358, *p* = 0.025) as well as the Post-noise group (*t* = 3.210, *p* = 0.003) at 60% MD.

Further, the masking effect difference between the two masker to the nfEFR (Figure 6F) was also examined by a two-way ANOVA. A significant group effect was found for group (F_1_ = 4.192, *p* = 0.049), but not for MD. However, the *post hoc* comparisons (Holm-Sidak method) revealed no significant difference between groups within either of the MDs (30% MD; *t* = 1.330, *p* = 0.193, 60% MD; *t* = 1.566, *p* = 0.127).

### Transient Compound Action Potential and Contralateral Suppression

**FIGURE 7:**
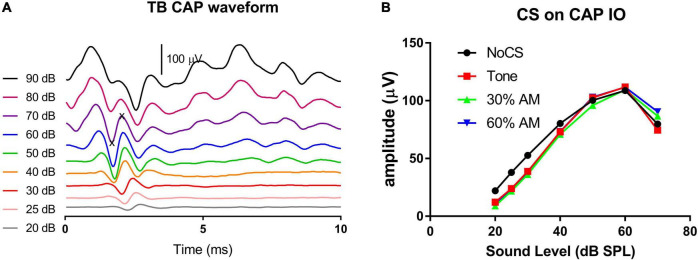
CAP waveforms across sound levels **(A)** and the exemplary CS effect on CAP I/O functions **(B)**. Three CS signals (tones, AM with 30% and 60% modulation depths, respectively) were all presented at 75 dB SPL. They show a similar CS effect, which were larger at lower levels of tone bursts evoking CAP. The CAP amplitude was measured between “x” symbols **(A)**.

The impact of modulated auditory input on medial olive cochlea (MOC) efferent control was observed *via* contralateral suppression on transient CAP, which was measured in response to 16 kHz tone bursts. Figure 7A shows CAP waveforms from one subject at levels from 90 to 20 dB SPL. The peak-to-peak value was read from the first negative peak to the next positive peak. Since the CAP was contaminated by the summating potential at sound levels above 70 dB SPL, the input/output (I/O) function was measured up to this level. Figure 7B shows the typical CS effect on an exemplary CAP I/O function. The suppression effected by the three CS signals was quite similar and was larger at lower levels of CAP-evoking tone bursts.

**FIGURE 8:**
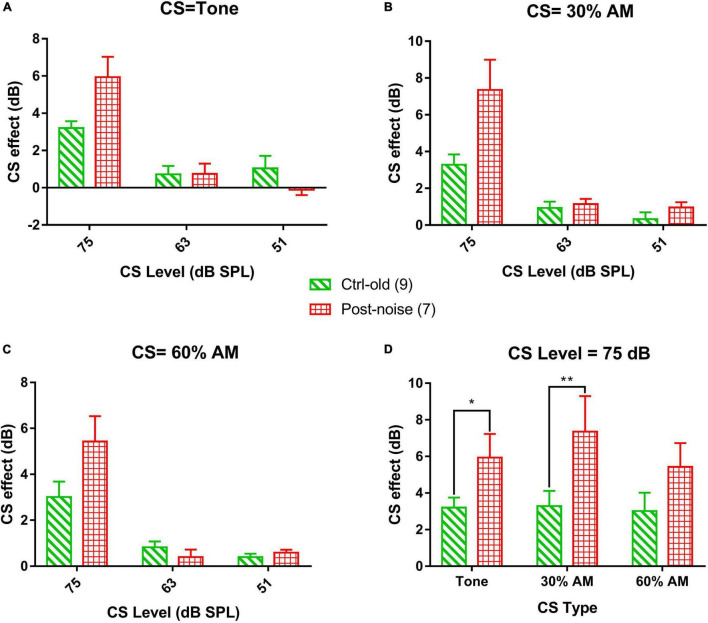
The CS effect on transient CAP in response to 16 kHz. tone bursts at the low-level average (the average of the stimulation levels of 20, 25, and 30 dB SPL). The CS signals are 16 kHz tone bursts, and AM with 30% and 60% modulation, respectively, across three levels (75-, 63- and 51-dB SPL). **(A–C)** The effect of CS level on the CAP across groups, showing a decreased CS effect with decreasing CS level, consistent across the three types of CS signals. **(D)** Comparison of CS effect across CS type and group with 75 dB SPL CS signals.

The CS effect was calculated in dB using the formula 20log[(CAP without CS)/(CAP with CS)]. Since the CS effect was larger at lower levels, the low-level average was calculated across the 30, 25, and 20 dB SPL tone bursts levels. Figures 8A–C show the CS effect caused by each of the three CS signal types (16 kHz stationary tone, and the same tone amplitude modulated at 30% and 60% MD). For each stimulus, CS effects were measured at three CS signal levels (75, 63 and 51 dB SPL). Overall, three trends can be seen for the CS effect across level and type of CS signal: (1) a larger CS effect is seen with a higher CS level, with no exception for the modulated CS signal (AM tone) as we hypothesized by the stronger fluctuation in HSR ANFS in response to a low sound level, (2) there is no obvious difference in the CS effect across the CS signal types, (3) CS effects were not reduced but rather increased in the noise group; suggesting that NIS did not impair MOC regulation on cochlear gain. Since the CAP suppression by the two lower CS signals was very small, further analysis focused only on the CS effect produced by the CS signal at 75 dB SPL to show the potential impact of CS type and group (Figure 8C). A two-way ANOVA performed for this purpose revealed a significant effect of group (*F* = 18.823, *p* < 0.001) but no significant effect of CS type (*F* = 1.747, *p* = 0.199). *Post hoc* comparisons were then performed (Holm-Sidak method) and revealed a significant difference between the Ctrl-old and the Post-noise groups within tone bursts signal type (*t* = 2.227, *p* = 0.031) and within the 30% AM signal type (*t* = 3.316, *p* = 0.002).

## Discussion

In the present study, the noise exposure was similar to that occurring in human experience in terms of level and temporal features and was applied at a lower level (around 90 dB SPL). The permanent reduction of synaptic density in the high frequency region was only 6% (Figure 3)— much lower than our previous reports after noise exposure at a high sound level in Guinea pigs (106 dB SPL, 2 h). We applied the noise exposure for 122 h to make the total energy of this exposure equivalent to that of the brief noise exposure at higher levels (100–106 dB SPL) used in previous studies that found a massive loss of afferent synapses in rodent cochleae (Kujawa and Liberman, 2009; Liu et al., 2012; Furman et al., 2013; Shi et al., 2013; Song et al., 2016; Chen et al., 2019b; Fan et al., 2020; Zhang et al., 2020). The equal-energy hypothesis is a rule of thumb, which states that noise exposure with equal energy should produce an equal amount of damage or NIHL even if presented at a different intensity level, at least after adjusting for kurtosis. This rule has been supported by many researchers in the field (Ward et al., 1981; Gomez Estancona et al., 1983; Lindgren and Axelsson, 1983; Roberto et al., 1985; Fredelius et al., 1987; Borg and Engstrom, 1989; Qiu et al., 2007). However, the results of the present study suggest that noise-induced synaptic loss is an exception to this rule. This is consistent with a previous report in which much less synaptic loss was found in CBA mice after a continuous noise exposure for 168 h at 84 dB SPL (Maison et al., 2013) as compared with a previous report using a more common brief noise exposure (100 dB SPL, 2 h) on the same strain of mice. The brief noise exposure yielded less total energy than the 168-h exposure at 84 dB SPL but produced 50% more synaptic loss in the high frequency region (Kujawa and Liberman, 2009).

Coding-in-noise deficits have been thought to be the most likely functional hearing difficulty associated with NIS without PTS (Plack et al., 2014; Le Prell and Clavier, 2017; Liberman, 2017; Liberman and Kujawa, 2017; Chen et al., 2019a; Huet et al., 2019; Kohrman et al., 2020). While great efforts have been made to verify the existence of CIND after NIS, results have been equivocal. To the best of our knowledge, only one animal study has found positive evidence for CIND after noise exposure, which was done in rats (Lobarinas et al., 2017). However, this study did not measure synaptic loss and several technical limitations make it difficult to interpret the result [see our review (Chen et al., 2019a) for details]. In human studies, reports with negative results (Fulbright et al., 2017; Grinn et al., 2017; Grose et al., 2017; Le Prell and Clavier, 2017; Prendergast et al., 2017a,^2019^; Yeend et al., 2017; Guest et al., 2018, 2019; Valderrama et al., 2018) have been more plentiful than those with positive results (Alvord, 1983; Kujala et al., 2004; Stone et al., 2008; Kumar et al., 2012; Stamper and Johnson, 2015; Liberman et al., 2016; Tepe et al., 2017; Meehan et al., 2019). The variability in results could be partially rooted in methodological error or measurement inconsistency, including imprecise quantification of noise exposure based on different types of self-report, a lack of objective measurement of synaptic loss or its functional consequences, and different approaches to measuring CIND [see our recent review for detail (Ripley et al., 2022)]. However, the lack of robust evidence for the CIND expected to occur with NIS and NIHHL should cause us to question the theoretical foundation on which this expectation is based, which assumes a unique rule for LSR ANFs for coding signals in high-level background noise. In a recent review, this assumption has been challenged systematically (Carney, 2018).

While CIND remains to be proved to be the major functional difficulty associated with NIHHL, temporal processing disorders have been verified in subjects with (potential) auditory neuropathy including NIS (Bharadwaj et al., 2014, 2015; Shaheen et al., 2015; Mehraei et al., 2016; Prendergast et al., 2017b,^2019^; Parthasarathy and Kujawa, 2018). It is reasonable to expect a deterioration in temporal processing ability after synaptic damage in the cochlea considering the function and importance of ribbon synapses in temporal processing. We have demonstrated this deficit in a single unit study in Guinea pigs with NIS (Song et al., 2016). Such temporal processing deficits may explain signal processing difficulties in noise, since poorer word scores tested with background babble have been found in conjunction with poorer temporal resolution as evaluated *via* gap-detection (Snell et al., 2002). In studies of NIHL, poorer temporal processing has also been correlated with noise-exposure history (Stone et al., 2008) and poorer speech perception in noise (Kumar et al., 2012).

In the present study, temporal processing was assessed *via* EFR TMTFs measured in both far field (EFR) and near field (nfEFR). A deterioration in temporal processing would be evident if the response amplitude was reduced, specifically shown as a sharper drop with increasing modulation frequency. Temporal processing deficits were found in a reduction of far field EFR at high modulation frequencies in the Post-noise group as compared to the Ctrl-old group. This was seen at the 30% MD only (Figure 4A). However, the TMTFs were largely overlapping between groups in nfEFR, suggesting a central origin of the changes in far-field EFR. A noise-induced change in far-field EFR TMTF has been reported previously in mice (Shaheen et al., 2015). In this study, band-pass TMTFs were reported with a peak close to a 1000 Hz MF. The ANF origin of this peak response was supported by its disappearance (or reduction) in the test after NIS was established. In the present Guinea pig study, however, the TMTFs showed a low-pass characteristic. The impact of noise was evidenced by a reduction of TMTFs at higher MFs but only when measured with AM at a 30% MD. A shallow MD has been recommended since it should be more sensitive to NIS which might be limited or biased to synapses connecting inner hair cells with low and medium SR ANFs (Bharadwaj et al., 2014). This is supported by the differences in TMTFs measured at two MDs in the present study (Figures 4A,B). In addition to the species difference, the EFRs in Shaheen’s study were evaluated with an AM signal at 100% MD, and the NIS was more severe than in the present report (Shaheen et al., 2015).

One of the most common ways to evaluate hearing in noise is to test signal perception or coding with masking. To evaluate if the signal coding in noise depends on temporal processing, the masker should be temporarily modulated to allow for signal detection in the temporal dips of the masker. However, this technical matter has received little attention (Souchal et al., 2018; Chen et al., 2019b; Ralli et al., 2019; Zhang et al., 2020). In the present study, we compared the effects of a stationary masker with those of a modulated masker. We hypothesized that if NIS reduces the ability to detect a signal in the dips of a masker, the masking effect should be greater with a modulated masker, such that the differences between the two maskers should be decreased. However, this hypothesis was not supported by our results. The masking effect on the EFR by both maskers was not larger in the Post-noise group than the Control group (Figure 6A), and there was no difference between groups in EFR as a function of masker type (Figure 6C). Interestingly, there was a significant difference between the two maskers in nfEFR, but the difference between the maskers was larger in the post-noise group, in opposition to our hypothesis. Therefore, the present study does not provide clear evidence for NIS-related deterioration in signal detection in noise *via* using temporal cues. It is important to mention that in the present study, the amplitudes of nfEFR were reduced in the Post-noise group (shown in Figures 6D,E as the larger attenuation in the Post-noise group). This is consistent with our previous studies using high level noise exposures (Chen et al., 2019b; Zhang et al., 2020), although the synaptic loss in the present study was much less.

An alternative model of signal coding at high sound levels was proposed by Carney after challenging a unique role for LSR ANFs in this process (Carney, 2018). The so-called fluctuation profile model is specific for the coding of complex signals like speech at high levels *via* HSR ANFs. In voiced speech, the amplitude of the signal is modulated at the fundamental frequency, and these temporal stimulus fluctuations modulate the firing rates of ANFs. At average speech levels (65–70 dB SPL), fluctuations in ANF firing rate are expected to be minimal or absent at formant peaks because these ANFs are saturated due to the high stimulus sound level. However, HSR ANFs in spectral troughs are not saturated and thus have strongly modulated firing patterns. Therefore, the distribution of temporal fluctuations of HSR ANF firing rates across frequency provides a profile that mirrors the spectrum of the speech. Extending from this theory, Carney proposed that temporal fluctuations in neural firing in the ascending auditory pathway may play an important role in controlling efferent feedback *via* medial olivo-cochlear neurons (MOCN). Temporal fluctuations in ANF responses are inherited by the cochlear nucleus and inferior colliculus (Gummer et al., 1988; Krishna and Semple, 2000; Joris et al., 2004; Carney et al., 2016). Carney suggested that sensitivity to such temporal fluctuations in the inferior colliculus may have an important role in regulating cochlear gain *via* the descending pathway from inferior colliculus to MOCN, providing a mechanism for enhancing fluctuation profile contrast. This is because ANFs near formant peaks show minimal to no fluctuation in firing rate (due to saturation) and therefore produce less excitation at the inferior colliculus and MOCN, resulting in less gain reduction than at frequencies near formant troughs. If this is correct, a modulated stimulus (such as an AM signal) should produce a stronger gain reduction *via* MOC feedback.

The efferent control to the cochlea is divided into two parts: (1) lateral efferent control from efferent neurons surrounding the lateral superior olive to the terminals of the type I afferent neurons under inner hair cells; (2) medial efferent control from MOCNs to the bodies of outer hair cells. The function of MOC control is much better understood as to regulate the active gain of outer hair cells, which in turn changes the response of ANFs to sound. While the pathway and function of efferent control *via* the lower brainstem have been comprehensively explored, corticofugal control from higher level auditory centers (such as the inferior colliculus) are less understood [see reviews (Terreros and Delano, 2015; Guinan, 2018; Fuchs and Lauer, 2019)].

MOC feedback is usually examined using a CS paradigm *via* otoacoustic emissions (OAE) or CAP. However, in most studies, CS signals are not temporarily modulated. In studies evaluating the effect of CS signal level, suppression has been found to be larger at higher CS signal levels [e.g., (Moulin et al., 1993; Zhang et al., 2007) for OAE and (Puria et al., 1996) for CAP]. In the present study, we used CAP to compare the amount of CS achieved with a stationary versus modulated suppressor signal. We predicted that, if temporal fluctuation is critical in MOC feedback, the modulated CS would produce a larger CS effect, which would be more so if presented at a relatively low level because HSR ANFs are not saturated at this level. However, our results did not show significant differences between the stationary and modulated suppressor signals with respect to CS. Moreover, the CS effect was always greater at a higher CS level, regardless of CS types. This negative result may not be adequate to fully reject a dominant role of temporal fluctuation of HSR ANFs in modulating MOCN-mediated efferent control. It is likely that a feedback loop relying upon average rate, rather than fluctuation, also exists (Carney, 2018), and these would have opposing effects. A role for stimulus fluctuation in MOC efferent control may therefore be difficult to detect.

The role of MOC efferent control in the development of NIS has been verified in mice: the degree of NIHHL was found to be positively correlated with the activity level of cholinergic receptors that were regulated by genetic manipulation (Boero et al., 2018, 2020). However, it is not clear if noise exposure itself can change MOC control of outer hair cells. Overall, our results did not show a reduction of CS to CAP in the noise group. Instead, there was an enhancement of the CS effect. Further studies are needed to confirm this enhancement in subjects with a larger amount of synaptic damage and loss.

In conclusion, the present study demonstrated that modulated, intermittent noise exposure common in real life is less effective in causing NIS. The risk of NIS without PTS and NIHHL may thus be lower than previously thought. With the smaller amount of NIS established by the noise exposure in this study, degradations in signal processing were likely limited and not reflective of those occurring with more severe NIS and NIHHL. Interestingly, while temporal processing dysfunction was seen in the far-field EFR TMTF, corresponding changes were not shown *via* nfEFR, suggesting a central origin for the changes in temporal processing. In contrast, a greater effect of masking on the EFR with NIS was only found in near-field measures, suggesting a peripheral origin for this effect along with central compensation. This result devalues the usefulness of EFR in evaluating the coding deficits associated with NIS. Furthermore, the temporal processing dysfunction did not appear to be related to the masking effect, given the different origins and the lack of any significant difference between the masking effect found with a stationary versus modulated masker. Finally, the results were not supportive of a role of temporal fluctuation in the MOC efferent control on cochlear gain.

## Data Availability Statement

The raw data supporting the conclusions of this article will be made available by the authors, without undue reservation.

## Ethics Statement

The animal study was reviewed and approved by Dalhousie University Committee of Laboratory Animals.

## Author Contributions

SR, XY, and SA: conceptualization, visualization, and writing. LX and JW: conceptualization, visualization, writing, and funding acquisition. All authors contributed to the article and approved the submitted version.

## Conflict of Interest

The authors declare that the research was conducted in the absence of any commercial or financial relationships that could be construed as a potential conflict of interest.

## Publisher’s Note

All claims expressed in this article are solely those of the authors and do not necessarily represent those of their affiliated organizations, or those of the publisher, the editors and the reviewers. Any product that may be evaluated in this article, or claim that may be made by its manufacturer, is not guaranteed or endorsed by the publisher.

## Abbreviations

ABR: auditory brainstem response
AM: amplitude modulation
ANF: Auditory nerve fibers
CtBP2: C-terminal binding protein 2
CAP: compound action potential
CIND: coding-in-noise deficit
CS: contralateral suppression
EFR: envelop following responses
L/M/HSR ANF: low/medial/high SR ANF
MD: modulation depth
MF: modulation frequency
MOCN: Medial olive-cochlea neurons
nfEFR: near-field EFR
NIS: noise induced synaptopathy
NIHHL: noise induced hidden hearing loss
SR: spontaneous rate
PSD: post-synaptic density
TMTF: temporal modulation transfer function.
