# Supplementary material for: Synaptopathy in Guinea Pigs Induced by Noise Mimicking Human Experience and Associated Changes in Auditory Signal Processing
Source: Front Neurosci. 2022 Jul 6;16:935371. doi: 10.3389/fnins.2022.935371 (PMC9298651; doi:10.3389/fnins.2022.935371)
Supplement: Supplementary file 1 [file Data_Sheet_1.docx]

Supplementary materials:

To do frequency shifting of multi-talker babble, a zero-phase 1000-point finite impulse response (FIR) filter was used to filter the multi-talker babble into 19 third-octave bands with centre frequencies ranging from 125 Hz to 8 kHz. Then, a white noise was filtered into 19 sixth-octave bands from 2 to 16 kHz. In each speech band, the amplitude envelope was extracted by calculating the absolute value of the Hilbert transform of the filtered band. Each speech envelope was then bandpass filtered between 2 and 50 Hz to eliminate envelope fluctuations outside of the range of frequencies that reflect human articulatory movements. Next, from each third-octave speech band, the filtered envelope was used to modulate the corresponding sixth-octave noise band. The sixth-octave filter that was used to extract the noise prior to modulation was then used to refilter each modulated noise band. The purpose of this step was to remove any out-of-band energy introduced by the envelope shaping. Then, each noise band was scaled to have the same root-mean square (RMS) amplitude as the speech band from which their modulations were derived. Finally, the 19 modulated noise bands were summed to produce the modified 2-16 kHz multi-talker babble).
